# Analysis of Human Clinical Mutations of Mitochondrial ND1 in a Bacterial Model System for Complex I

**DOI:** 10.3390/life12111934

**Published:** 2022-11-20

**Authors:** Hind A. Alkhaldi, Duong H. Phan, Steven B. Vik

**Affiliations:** Department of Biological Sciences, Southern Methodist University, Dallas, TX 75275, USA

**Keywords:** Complex I, mitochondria, bioenergetics, NADH dehydrogenase, mutations, cardiomyopathy, LHON

## Abstract

**Simple Summary:**

Genetic diseases can now be detected directly by determining the DNA sequence of genes, through recent advances in technology. Of the more than 20,000 human genes, several dozen are located in the mitochondria of cells, rather than the nucleus. These genes are essential for energy production and have the unique feature of being passed directly from the mother to her children. After identification of mutations in mitochondrial genes of patients, it is not always simple to determine if the disease is caused by the identified mutation. In this study we have analyzed nine mutations in a mitochondrial gene called ND1 that were reported in the medical literature. We have modeled those mutations in a closely related gene found in bacteria, because it is currently not possible to do this in human cells. We confirmed that five of the mutations cause significant loss of function in the bacterial system, consistent with a severe disease state in humans. The other four mutations had milder effects, and this suggests that additional factors might be necessary for severe disease in humans. Such information can be useful in genetic counseling of women carrying mitochondrial mutations.

**Abstract:**

The most common causes of mitochondrial dysfunction and disease include mutations in subunits and assembly factors of Complex I. Numerous mutations in the mitochondrial gene ND1 have been identified in humans. Currently, a bacterial model system provides the only method for rapid construction and analysis of mutations in homologs of human ND1. In this report, we have identified nine mutations in human ND1 that are reported to be pathogenic and are located at subunit interfaces. Our hypothesis was that these mutations would disrupt Complex I assembly. Seventeen mutations were constructed in the homologous *nuoH* gene in an *E. coli* model system. In addition to the clinical mutations, alanine substitutions were constructed in order to distinguish between a deleterious effect from the introduction of the mutant residue and the loss of the original residue. The mutations were moved to an expression vector containing all thirteen genes of the *E. coli nuo* operon coding for Complex I. Membrane vesicles were prepared and rates of deamino-NADH oxidase activity and proton translocation were measured. Samples were also tested for assembly by native gel electrophoresis and for expression of NuoH by immunoblotting. A range of outcomes was observed: Mutations at four of the sites allow normal assembly with moderate activity (50–76% of wild type). Mutations at the other sites disrupt assembly and/or activity, and in some cases the outcomes depend upon the amino acid introduced. In general, the outcomes are consistent with the proposed pathogenicity in humans.

## 1. Introduction

Mitochondrial Complex I (NADH:ubiquinone oxidoreductase) is the largest member of the mammalian electron transport chain. It contains 45 protein subunits, one flavin mononucleotide (FMN) and eight FeS clusters. Thirty-eight of these subunits, one in two copies, are encoded by 37 nuclear genes, and these proteins are imported into the mitochondrion. The remaining seven subunits are encoded by mitochondrial genes, ND1-6 plus ND4L [1,2]. Mutations in these genes are known to be involved in many mitochondrial diseases [3,4,5], including Leber’s Hereditary Optic Neuropathy (LHON) [6], mitochondrial encephalopathy, lactic acidosis and stroke-like episodes (MELAS) [7], cardiomyopathies [8], and Leigh syndrome [9].

There are three essential activities of Complex I that are found in all mitochondrial and bacterial versions of the enzyme and are carried out by fourteen core subunits: NADH oxidation, ubiquinone reduction, and proton translocation. NADH oxidation regenerates NAD^+^ for the citric acid cycle and reduces the flavin. Electrons that pass through the flavin eventually reduce ubiquinone, which drives proton translocation. The reduced quinone will be re-oxidized by Complex III, or other enzymes, resulting in additional proton translocation. The functions of electron transport and proton translocation are carried out by two distinct regions of Complex I, sometimes referred to as the peripheral and membrane arms. The seven mitochondrially-encoded subunits are found in the membrane arms. The seven nuclearly-encoded core subunits are found in the peripheral arm of Complex I, which extends into the mitochondrial matrix, or the cytoplasm in the case of bacteria. A key subunit, ND1, is found at the junction of the two arms, and is essential for both reduction of the quinone and the initiation of proton translocation. ND1 helps to form the quinone-binding site near the terminal FeS cluster, N2. It also works with ND3 for energy transduction to drive proton translocation by undergoing conformational changes in response to quinone reduction. (For recent reviews of Complex I see [10,11,12,13,14]).

Advances in DNA sequencing have allowed detection of ever-increasing numbers of mutations in mitochondrial genes. Such mutations are tabulated at websites such as MitoMap (www.mitomap.org, accessed on 1 July 2022) [15] and ClinVar (https://www.ncbi.nlm.nih.gov/clinvar/, accessed on 1 July 2022) [16], along with an evaluation of whether the mutation is considered pathogenic. It can be difficult to ascertain whether an identified mutation is causative for disease. Biochemical analyses of mitochondrial function from tissue samples are helpful but are not always available. Moreover, control values of enzyme activities are typically uncertain and are specified by a broad range. The high copy number of mitochondrial DNA in most cells allows heteroplasmy of a mutation to vary from 0 to 100%, and this value typically varies from one cell type to another. To further complicate the matter, the mitochondrial haplotype and the broader genetic background of the individual appear to influence the effect of a particular mutation, and generally in unknown ways [17]. For all these reasons, the pathogenicity of an identified mitochondrial mutation can be difficult to establish [18,19,20].

Currently it is still challenging to construct and analyze mutations in mitochondrial genes. Cybrid cells, in which mitochondria with existing mitochondrial mutations are transferred to cells lacking mitochondria, have been used to confirm pathogenicity of mutations in mitochondrial genes [21]. New gene editing techniques hold promise, such as the recently described base-editing method [22]. Another approach to is to model the mutations in a bacterial system. An early example of this is the use of *P*. *denitrificans*, a bacterium with proteins closely related to human mitochondrial ones, in which mutations found at residue 52 in ND1 that cause LHON were examined [23]. The authors suggested that this residue is at the ubiquinone-binding site. A recent porcine structure of Complex I with bound ubiquinone supports that interpretation [24].

**Figure 1:**
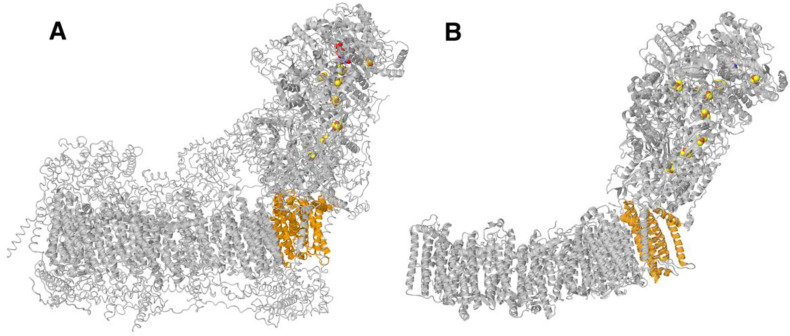
Comparison of the structure of human and *E. coli* Complex I. (**A**) Human (PDB ID: 5XTD). (**B**) *E. coli* (PDB ID: 7NYR). Core subunits are shown in ribbons, while supernumerary subunits of the human enzyme are shown in backbone trace. Subunits shown in orange color are those that contain clinical mutations, ND1 (**A**) or NuoH, which has been mutated in this study (**B**). FeS clusters and FMN are shown in space-filling view.

In this report, we examine mutations in ND1 of human Complex I using an *E. coli* model system. *E*. *coli* contains 13 genes in the *nuoA-N* operon that code for the 14 core subunits (genes *nuoC* and *nuoD* are fused in some bacterial species). See Figure 1 for a comparison of the human and *E. coli* enzymes. In recent years, numerous high-resolution structures of Complex I from mitochondria [25,26,27,28,29,30,31,32,33] have been determined. Furthermore, several new bacterial structures have been published [34,35,36]. We have selected mutations in ND1 that map to subunit interfaces and modeled them in the *nuoH* gene of *E. coli*. The mutated genes can be expressed from an arabinose-induced pBAD plasmid containing all Complex I genes, in a host strain deleted for the *nuo* operon, allowing for simple comparison of mutants [37]. In general, a second mutation was also constructed, changing the residue to alanine. Results indicated whether loss of function is associated with the loss of the original amino acid, or by the introduction of the mutant amino acid. We used antibodies to determine expression level by tagging NuoH with HA and native gels to determine the assembly state. In earlier studies of bacterial Complex I antibodies to subunit H, high-resolution structures were not available. In general, the outcomes here were consistent with the observed or predicted pathogenicity in humans.

## 2. Materials and Methods

### 2.1. Materials

Restriction endonucleases, T4 DNA ligase, Q5 Site-Directed Mutagenesis Kit, Q5 High-Fidelity PCR Kit, and Monarch PCR Cleanup Kit were from New England Bio Labs (Beverly, MA, USA). MauBI restriction endonuclease and DNA Miniprep kits were from ThermoFisher Scientific (Pittsburgh, PA, USA). QIAquick and QiaIIX Gel Extraction Kits were from Qiagen (Germantown, MD, USA). Oligonucleotides for plasmid construction, mutagenesis, and sequencing were synthesized by Eurofins Genomics (Louisville, KY, USA). Immunoblot polyvinylidene difluoride (PVDF), Mini-PROTEAN TGX gels (12%, 4–15%), Precision Plus Protein ™ Dual Color standards, Precision Plus Protein™ unstained standards and the DC protein assay kit were from Bio-Rad (Hercules, CA, USA). 9-Amino-6-chloro-2-methoxyacridine (ACMA) and NativeMark™ Unstained Protein Standards were from Invitrogen. The polyclonal antibody against *E. coli* Complex I subunit CD was a generous gift from T. Yagi and A. Matsuno-Yagi (Scripps Research Institute, La Jolla, CA, USA). Subunits H and N were detected by the monoclonal mouse HA-probe (F7), from Santa Cruz Biotechnology (Dallas, TX, USA), as were goat anti-rabbit and goat anti-mouse IgG-HRP. SuperSignal West Dura Extended Duration Substrate and *p*-nitro blue tetrazolium chloride (NBT) were from ThermoFisher Scientific (Pittsburgh, PA, USA). *n*-dodecyl-β-D-maltopyranoside (dodecyl maltoside) was from Anatrace (Maumee, OH, USA). Other chemicals, including NADH, 6-aminocaproic acid, L-arabinose, Coomassie Brilliant Blue G, Bis-Tris, deamino-NADH (dNADH), and carbonyl cyanide-*p*-trifluoromethoxyphenylhydrazone (FCCP) were from MilliporeSigma (Burlington, MA, USA).

### 2.2. Mutagenesis

Single amino acid substitutions were constructed by Q5 Site-Directed Mutagenesis with an additional restriction site introduced for rapid identification of mutants. Primers used for construction of mutations are found in Supplementary Table S1. Mutations were constructed in pBAD33(G-H•HA-I) (Alkhaldi and Vik-under revision) and transferred to the expression vector pBAD33(A-N), which contains a full size *nuo* operon [37] by using restriction sites for MauB I in *nuoG* and Kpn I in *nuoI*. The plasmids were then used to transform strain BA14, which carries a chromosomal deletion for the *nuo* genes of all Complex I subunits [38]. DNA sequencing to confirm the presence of mutations was performed by Lone Star Labs (Houston, TX, USA).

### 2.3. Plasmids and Strains

XLI-Blue (*recA*, *endA1 gyrA96 thi*-1 *hsdR17 supE44 relA1 lac* {*F′ proAB lacI^q^* Z∆M15 Tn10 (Tet^R^)}) was used for plasmid construction and propagation. BA14 (*bglR*, *thi*-1, *rel*-1, *HfrPO1*, ∆*nuoA-N*) with deletion of the entire *nuo* operon was used as the host for gene expression [38]. 10-Beta cells, a derivative of DH10B from England Biolabs Δ*(ara-leu) 7697 araD139 fhuA* Δ*lacX74 galK16 galE15 e14-*ϕ*80*d*lacZ*Δ*M15 recA1 relA1 endA1 nupG rpsL* (Str^R^) *rph spoT1* Δ*(mrr-hsdRMS-mcrBC)* were used for transformation of ligation products from large fragments, forming plasmids greater than 15 kb.

### 2.4. Growth and Membrane Preparation

For expression of *E. coli* Complex I subunits and subsets of Complex I genes, the cells were grown in a rich chemical media (0.5% yeast extract, 1% peptone, 15 mM Na_3_PO_4_, 10 mM Na_2_HPO_4_, 25 mM KH_2_PO_4_, 50 mM NH_4_Cl, 5 mM Na_2_SO_4_, 2 mM MgSO_4_, 50 mg/L riboflavin, 30 mg/L ferric ammonium citrate, and 0.5 mM L-cysteine) at 37 °C [39]. The cells were harvested then suspended in buffer (50 mM MES, 10 mM MgSO_4_, 25% Glycerol, pH 6.0), and passed through the French Press at 8000 psi. Cell debris and unbroken cells were removed by a low-speed spin at 8000 rpm for 15 min in a JA-20 rotor. The supernatant was then transferred into a smaller tube and spun at higher speed 45,000 rpm for 1 h at 4 °C in a Beckman Ti-50 rotor. The pellet was resuspended in 1 mL of the same buffer and centrifuged at 100,000 rpm for 1 h at 4 °C in a TLA100.2 rotor. Then, the pellet was resuspended in 1 mL of the same buffer and used in subsequent experiments or stored at −80 °C. Enzyme activity measurements were always made before freezing. Protein concentration was determined by the Bio-Rad DC Protein Assay, using bovine serum albumin as standard.

### 2.5. SDS Electrophoresis and Immunoblotting

An amount of 50 μg of membrane protein was run in 12% acrylamide gels at 150 V for 1 h and then transferred to PVDF membranes by applying 100 V for 1 h in transfer buffer (25 mM Tris, 192 mM glycine, 20% methanol, pH 8.3). The PVDF membrane was blocked overnight with 5% milk in TBS/Tween 20 (20 mM Tris, 500 mM NaCl, 0.05% Tween 20, pH 7.5) at 4 °C and washed 3 times for 5 min with TBS/ Tween 20 the next day. The PVDF membrane was incubated at room temperature for 2 h with primary antibody diluted 1:20,000 for subunit CD. Mouse anti-HA probe was diluted 1:20,000. After washing 3 times for 5 min with TBS/Tween 20, the blot was incubated with anti-rabbit IgG-HRP or anti-mouse IgG-HRP (diluted 1:10,000) for 1 h. After three more washings with TBS/Tween 20, the blot was treated with horseradish peroxidase (HRP) chemiluminescent substrates in a ChemiDoc Imaging System (Bio-Rad, Hercules CA). Images were exported by Image Lab software. Blots shown are representative of blots from 2–3 preparations.

### 2.6. Blue-Native Gel Electrophoresis and NADH Dehydrogenase Activity Assay

Blue-native gel electrophoresis was performed according to previous methods [40,41]. In brief, *E. coli* membranes equivalent to 800 μg of protein were resuspended in 750 mM aminocaproic acid, 50 mM Bis-Tris-HCl (pH 7.0), 0.1 mg/mL DNase, and 0.8% (*w*/*v*) dodecyl maltoside to a total volume of 240 μL. After incubation on ice for 30 min, the samples were centrifuged at 149,000× *g* for 10 min. The supernatants were recovered, and 2% (*w*/*v*) of Coomassie Brilliant Blue G in 1 M aminocaproic acid was added at a final concentration of Coomassie Blue of 0.08% (*v*/*v*). Fifty μg protein from the samples was loaded on a 4–15% gradient gel, and electrophoresis was performed in a 4 °C chamber at 100 V until entry of the protein sample into the stacking gel. After that, the cathode buffer, containing 0.02% of Coomassie Blue, was replaced by the cathode buffer without Coomassie Blue, and the electrophoresis was continued at 150 V until the tracking dye ran out. After electrophoresis, the gel was washed several times in 2 mM Tris-HCl (pH 7.5) before transfer to PVDF. For the NADH dehydrogenase activity assay, the gel was incubated in 2 mM Tris-HCl (pH 7.5) containing 2.5 mg/mL of NBT and 150 μM NADH for 40 min at room temperature. The reaction was terminated by 10% acetic acid and 50% methanol. The blots shown are representative of 2–3 preparations.

### 2.7. Deamino-NADH Oxidase Activity Assay

The activity assays were modified for the Epoch 2 plate reader (BioTek, Burlington, VT) from methods described previously [38,42]. To avoid oxidation by the alternative NADH oxidase in *E. coli* membranes, we used deamino-NADH (dNADH) [43]. The dNADH oxidase activity was assayed by using oxygen as a terminal electron acceptor. NADH oxidase activity assays were started with 0.25 mM dNADH (extinction coefficient 6.22 mM^−1^ cm^−1^). The dNADH oxidase activity assay was performed in 50 mM MOPS, 10 mM MgCl_2_, pH 7.3 at 20 °C, and the absorbance was monitored at 340 nm for 2 min. The uncoupler FCCP was added to a final concentration 1 μM from a 1 mM ethanol stock. The rate of dNADH oxidase from membranes of BA14/pBAD33(A-N) was about 1.0 μmol dNADH per min per mg protein. Proton translocation assays were conducted by measuring the fluorescence quenching of the acridine dye ACMA as a ∆pH indicator using excitation and emission wave lengths of 410 and 490 nm, respectively. Proton translocation assays were performed in 50 mM MOPS, 5 mM MgCl_2_, 50 mM KCl (pH 7.3) at 20 °C. One µM valinomycin was included to eliminate the buildup of a membrane potential during NADH oxidation, and ACMA was 1 µM. The reactions were initiated with dNADH (0.25 mM) and after about 2 min, the proton gradient was collapsed by the addition of FCCP (1 µM) from 1 mM ethanol stock. The rates of fluorescence quenching are not strictly quantitative, but they give an indication of the ability of Complex I to translocate protons relative to the leakiness of the membrane vesicles. The rates shown are representative of 2–3 preparations with 3–4 replicates each.

## 3. Results

### 3.1. Mutations of Residues That Contact Subunits NDUFS7 and NDUFS8 (NuoB and NuoI)

#### 3.1.1. Description of the Human Mutations

**Figure 2:**
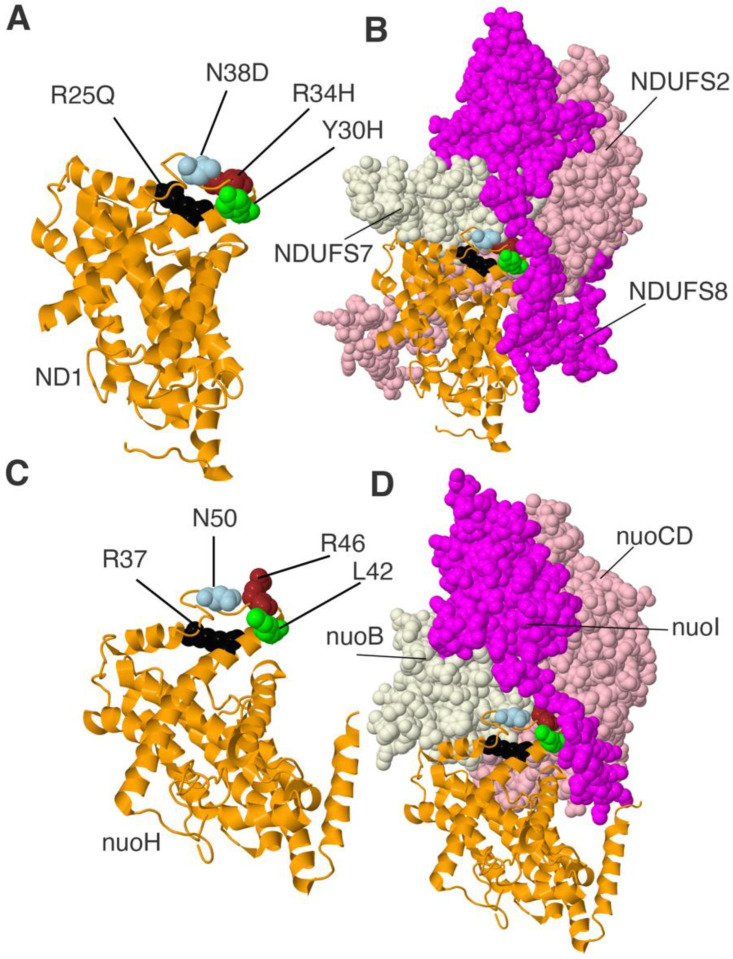
Modeling four human ND1 mutants in *nuoH* of *E. coli* Complex I. (**A**,**B**) ND1 (orange), NDUFS7 (beige), NDUFS2 (pink), and NDUFS8 (magenta) of human Complex I are shown. PDB ID: 5XTD. Clinical mutations are R25Q colored black, Y30H colored lime, R34H colored brown, and N38D colored light blue. (**C**,**D**) Subunit Nqo9 (NuoH) of *T. thermophilus* Complex I is shown in orange with Nqo4 (NuoCD) colored pink, Nqo6 (NuoB) colored beige, and Nqo9 (NuoI) colored magenta. PDB ID:4HEA. The four clinical mutants were modeled in the *nuoH* gene of *E. coli.* The corresponding residues in NuoH: R37 (black); R42 (lime); R46 (brown); and N50 (light blue).

Subunit ND1 in humans has 318 amino acids and a molecular mass of 36.7 kDa. It bears about 40% sequence identity to the core amino acids of NuoH from *E. coli* Complex I. This is considered to be sufficient for extensive structural similarity. Due to the recently obtained 3-dimensional structures of Complex I from various species including *E. coli*, this similarity can be confirmed for regions of interest. The ND1 subunit is found at the junction of the peripheral and membrane arms of Complex I. It contains eight tilted transmembrane helices, with matrix-facing loops that interact with core subunits NDUFS2 (NuoCD), NDUFS7 (NuoB), and NDUFS8 (NuoI). In the membrane, it interacts primarily with core subunit ND3 (NuoA). Four clinical mutations in ND1, R25Q, Y30H, R34H, and N38D were modeled in the *nuoH* gene as R37Q, L42S, R46H, and N50D. These residues are found on a matrix-side loop (R46 and N50) or the connecting transmembrane helix TM1 (R25 and Y30). The locations of these residues in the human and the *E. coli* proteins are shown in Figure 2. Because this region is not resolved in the recent structure from *E. coli* [34], the related protein from *Thermus thermophilus* is shown [44]. These residues are near subunit NDUFS7 (NuoB), except Y30, which contacts supernumerary subunits NDUFA1 (not shown) and NDUFS8 (NuoI).

The ND1_R25Q (G3380A) mutation was first identified in a 62-year-old woman with bilateral hearing loss since age 50 [45]. She later suffered vision loss and aphasia, and was diagnosed with MELAS. The mutation was heteroplasmic, with 50% levels in skeletal muscle and 5–10% in leukocytes. Complex I activity in skeletal muscle was in the normal range. ND1_Y30C (A3395G) was first reported in association with other mutations [46,47]. The ND1_Y30H mutant, similarly, was first reported with other mutations [48,49]. More recently, the Y30C mutation was studied in five individuals from three different families as an isolated mutation [50] with symptoms including deafness, exercise intolerance, and ataxia. Cybrid studies revealed a functional Complex I, but it was found expressed at a lower level. The ND1_R34H (G3407A) mutation was first reported in a 65-year-old male with hypertrophic cardiomyopathy [51], although later suggested to be a polymorphism of the M5a haplogroup [52]. The same mutation was found later among South African pediatric patients, but the activity of Complex I was not diminished [53]. The ND1_N38D (A3418G) mutation was reported in an individual with megakaryoblastic leukemia and was associated with high levels of superoxide production [54].

#### 3.1.2. Effect of R37, L42, R46, and N50 Substitutions on Function of Complex I

**Figure 3:**
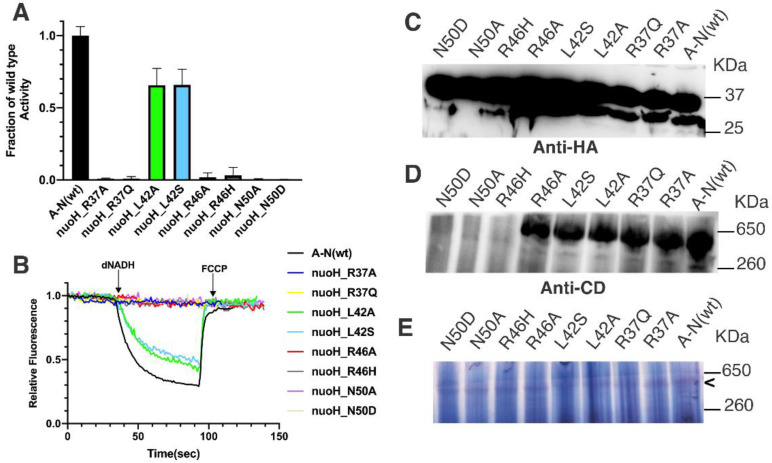
Effect of R37, L42, R46, and N50 substitutions in NuoH on activity of *E. coli* Complex I. NuoH_R37A,Q, L42A,S, R46A,H, and N50A,D were expressed from vector pBAD33(A-N), and membrane vesicles were prepared. (**A**) dNADH oxidase activity of the mutants were normalized to the value of activity from the wild type pBAD33(A-N). Results are the means and standard deviations from 2–3 membrane preparations with 4 replicates each. (**B**) Proton translocation activity of Complex I. (**C**) Samples from the previous panels were resolved by SDS gel electrophoresis, blotted, and probed by HA-antibody. Both NuoN (37 kDa) and NuoH (30 kDa) can be recognized, although NuoH is less sensitive. (**D**) Blue native gel electrophoresis was performed and immunoblotted using antibody against subunit CD. (**E**) I In-gel assay was performed after native gel electrophoresis. NADH dehydrogenase activity was shown by incubation with 2.5 mg/mL NBT and 150 µM NADH for 30 min. The band of Complex I activity is indicated by “<”.

In *E. coli*, R37A,Q, L42A,S, R46A,H, and N50A,D were constructed in *nuoH* and moved to the expression vector pBAD33(A-N). Membrane vesicles were prepared, and enzyme activity assays were measured. Complex I activities are shown in Figure 3: dNADH oxidase in panel A and proton translocation in panel B. The presence of NuoH is shown in panel C by immunoblotting of solubilized membranes. It should be noted that both NuoH and NuoN are tagged with HA, yielding bands of about 30 and 37 kd, respectively. In order to detect the NuoH signal, the NuoN must be overexposed. The presence of assembled Complex I is shown by native gel electrophoresis in panel D. Panel E shows the in-gel activity of the native Complex I.

The NuoH_L42A, and S mutants were the only ones of this group that had significant levels of activity, both about 66% of wild type. The levels of NuoH and assembled Complex I were normal as compared to wild type. The NuoH_R37A and Q mutants had essentially zero activity, while levels of NuoH and assembled Complex I were normal. The NuoH_N50A and D mutants also had near-zero activity, but membranes lacked NuoH, and so, also failed to assemble Complex I. The NuoH_R46A, and H mutants had very low levels of dNADH oxidase activity, both measuring less than 5% of the wild type rate. They differed in that R46A showed normal levels of NuoH and assembled Complex I, while R46H had low levels of NuoH and poor assembly of Complex I. All samples showed at least a weak band of in-gel activity after native gel electrophoresis, which includes a long incubation period, indicating at least a low level of assembly, even though it was not visible in the immunoblots (Figure 3).

### 3.2. Mutations of Residues That Contact Subunit ND3 (NuoA)

#### 3.2.1. Description of the Human Mutations

**Figure 4:**
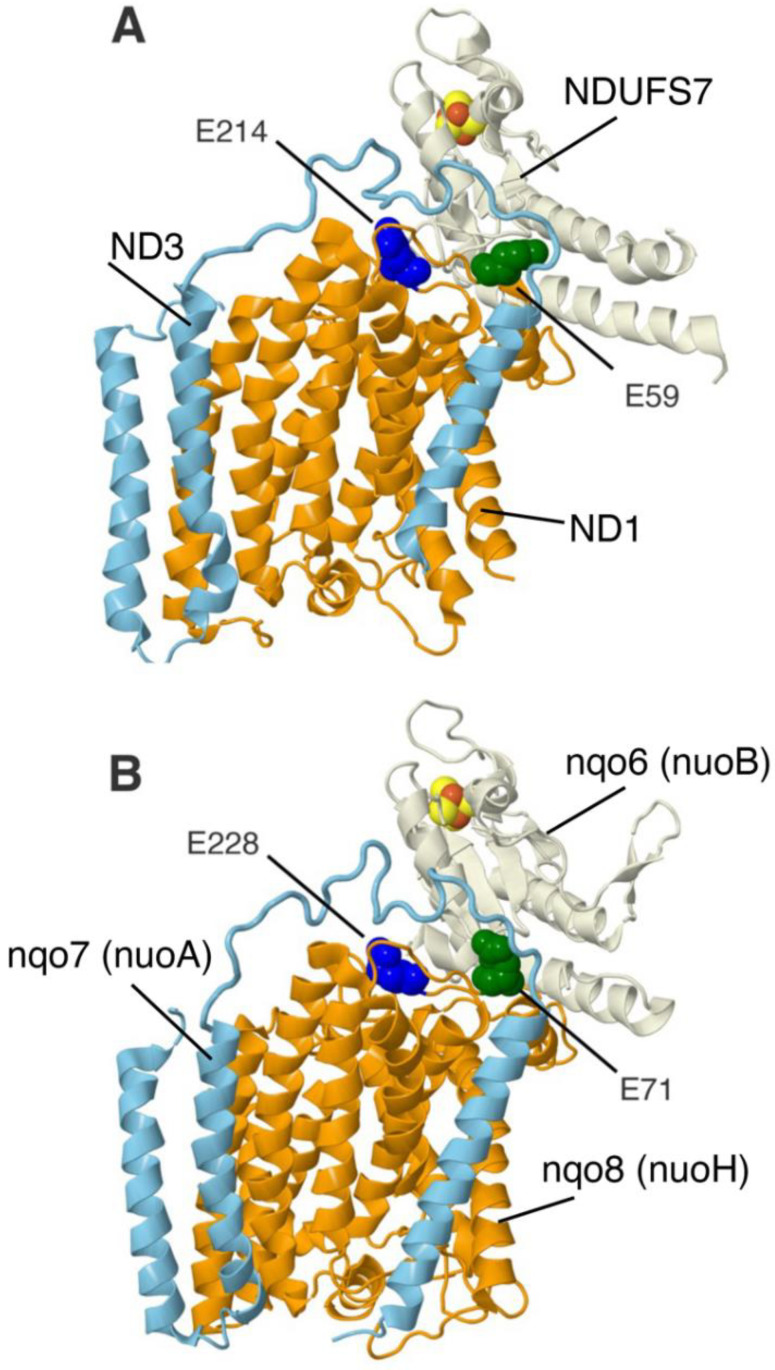
Modeling two human ND1 mutants in *nuoH* of *E. coli* Complex I. (**A**) ND1 (orange), NDUFS7 (beige), and ND3 (light blue) of human Complex I are shown. PDB ID: 5XTD. Clinical mutations are E59K (colored green) and E241K (colored blue). (**B**) Subunit Nqo8 (NuoH) of *T. thermophilus* Complex I is shown in orange with Nqo6 (NuoB) colored beige, and Nqo7 (NuoA) colored light blue. PDB ID:4HEA. The two clinical mutants were modeled in the *nuoH* gene of *E. coli.* The corresponding residues in subunit H: E71 (green) and E228 (blue).

Two clinical mutations in ND1, E59K, and E214K are each found in a different matrix-facing loop. They reside primarily between subunits NDUFS7 (NuoB), which contains the N2 FeS cluster, and ND3 (NuoA), which is a membrane-spanning subunit. They were modeled in the *nuoH* gene as E71K and E228K. The locations of the mutations for the human and the *E. coli* proteins are shown in Figure 4. Because the interacting region of NuoA is not resolved in the recent structure from *E. coli* [34], the related protein from *Thermus thermophilus* is shown [44].

#### 3.2.2. Effect of E71 and E228 Substitutions on Function of Complex I

**Figure 5:**
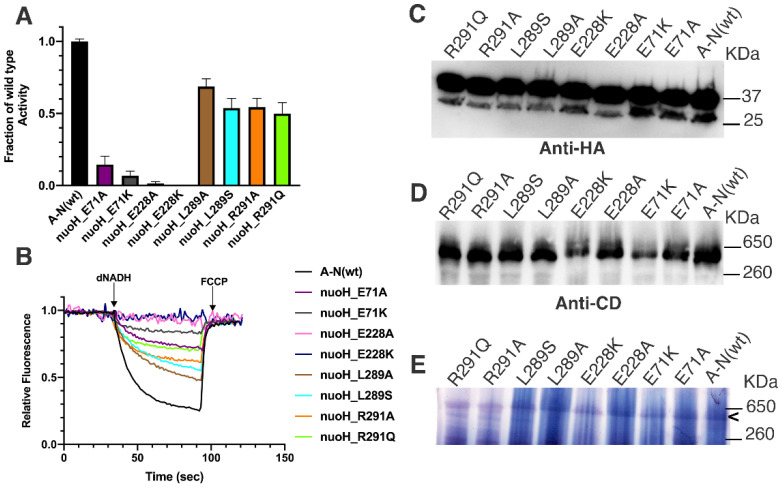
Analysis of E71, E228, L289, and R291 substitutions in NuoH of *E. coli* Complex I. NuoH_E71A,K, E228A,K, L289A,S, and R291A,Q were expressed from vector pBAD33(A-N), and membrane vesicles were prepared. (**A**) dNADH oxidase activity of the membranes is normalized to the value of activity from the wild type pBAD33(A-N). Results are the means and standard deviations from 2–3 membrane preparations with 4 replicates each. (**B**) Proton translocation activity of Complex I. (**C**) Samples from the previous panels were resolved by SDS gel electrophoresis, blotted, and probed by HA-antibody. Both NuoN (37 kDa) and NuoH (30 kDa) can be recognized, although NuoH is less sensitive. (**D**) Blue native gel electrophoresis was performed and immunoblotted using antibody against subunit CD. (**E**) In-gel assay was performed after native gel electrophoresis. NADH dehydrogenase activity was assayed by incubation with 2.5 mg/mL NBT and 150 µM NADH for 30 min. The band of Complex I activity is indicated by “<”.

In *E. coli*, E71A,K and E228A,K were constructed and moved to the expression vector pBAD33(A-N). Membrane vesicles were prepared, and enzyme activity assays were measured. Complex I activities are shown in Figure 5: dNADH oxidase in panel A and proton translocation in panel B. The rates of proton translocation (Figure 5B) corresponded to the rates of oxidase activity (Figure 5A). The presence of NuoH is shown to be similar to wild type for these mutants in panel C by immunoblotting of solubilized membranes. The presence of assembled Complex I is shown by native gel electrophoresis in panel D. Panel E shows the in-gel activity of the native Complex I. NuoH_E71 mutants have low activities, 15 ± 6% for E71A and 7 ± 3% for E71K, showing the especially deleterious effect of lysine. Assembly of Complex I was also more greatly reduced in NuoH_E71K, relative to the alanine substitution. The results for the E228 mutants were similar, but the activities were even lower. Activity of NuoH_E228A was barely detectable at about 1% of wild type, while E228K was essentially zero. Similar to the E71 mutants, NuoH_E228A showed a strong band of assembled Complex I (Figure 5D), while the band for E228K was much weaker. The in-gel activities (Figure 5E) appeared consistent with the immunoblots of Figure 5D.

### 3.3. Mutation of a Residue That Contacts NDUFS2 (NuoCD)

#### 3.3.1. Description of the Human Mutation

**Figure 6:**
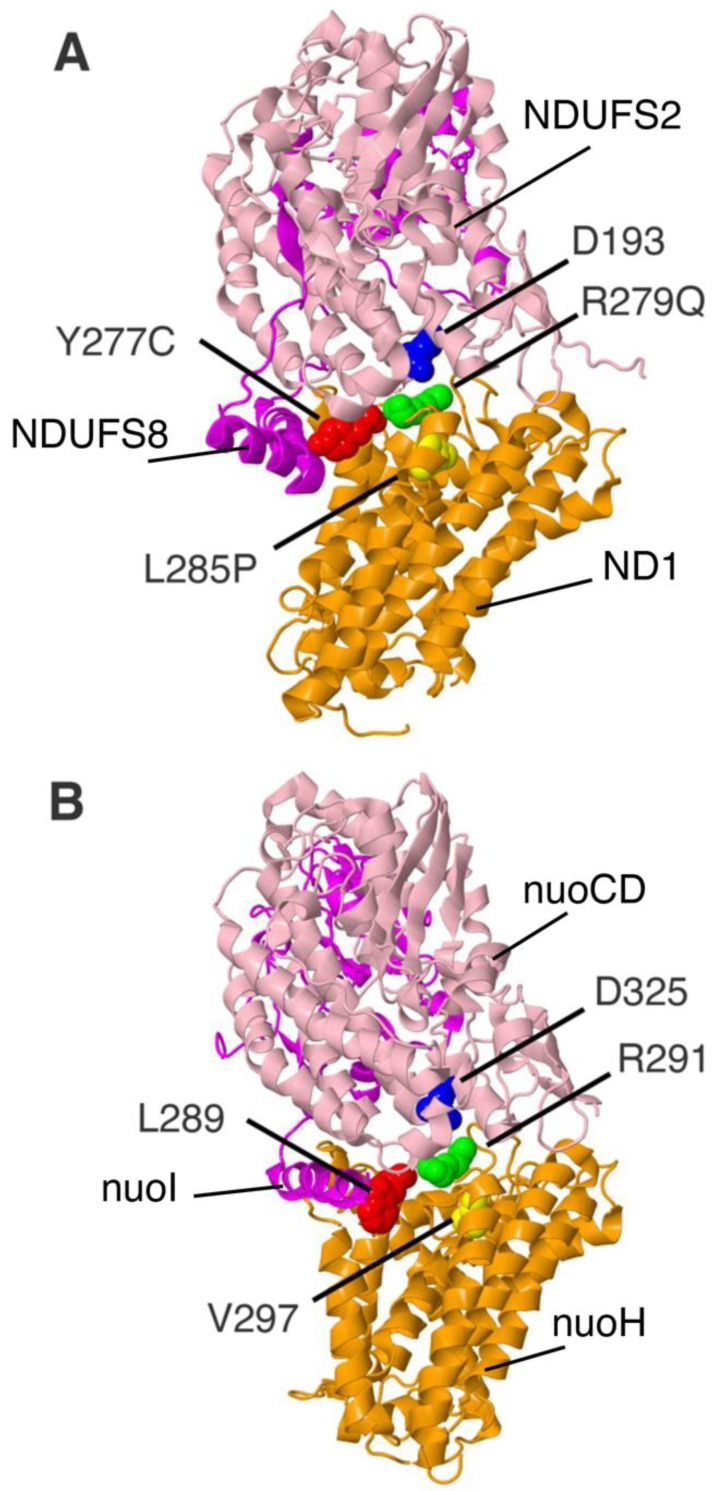
Modeling two human ND1 mutants in *nuoH* of *E. coli* Complex I. (**A**) ND1 (orange), NDUFS2 (pink), and NDUFS8 (magenta) of human Complex I are shown. PDB ID: 5XTD. Clinical mutations are Y277C colored red, L285P colored yellow, and R279Q colored lime. NDUFS2_D193 colored blue is in proximity to ND1_R279. (**B**) NuoH of *E. coli* Complex I is shown in orange with NuoCD colored pink and NuoI colored magenta. PDB ID: 7NYR. The *E. coli* residues of NuoH are L289 colored red, V297 colored yellow, R291 colored lime, and from NuoCD, D325 colored blue.

Residue R279 in ND1 is found in contact with NDUFS2, which does not contain any FeS clusters, but does contribute to the ubiquinone-binding pocket. Residue R279 is in proximity to a conserved aspartic acid, 193 in humans, and they do not appear to be engaged in an ion pair. R279 is hydrogen-bonded to the side chain of S205 of ND1. R279A and Q were modeled in the *nuoH* gene as R291A and R291Q. The locations of these residues for the human and the *E. coli* proteins are shown in Figure 6. The ND1_R279Q (G4142A) mutation was discovered in a 10-year-old by a Next Generation Sequencing screen [55]. The mutation was heteroplasmic: 69% in blood and 86% in muscle, while absent in the mother. The individual experienced developmental delay, seizures, and hypotonia.

#### 3.3.2. Effect of R291 Substitutions on Function of Complex I

In *E. coli*, R291A,Q were constructed in *nuoH* and moved to the expression vector pBAD33(A-N). Membrane vesicles were prepared, and enzyme activity assays were measured. Complex I activities are shown in Figure 5A. The rates of dNADH oxidase were nearly identical: NuoH_R291A was 55 ± 6% and R291Q was 50 ± 7%, indicating that loss of arginine was the major effect. The rates of proton translocation also appeared to be about 50% of wild type (Figure 5B). Both showed a band for NuoH in the immunoblot (Figure 5C). In the native gels (Figure 5D,E), both were similar to the wild type.

### 3.4. Mutations of Residues in ND1 Previously Proposed to Be Interacting

#### 3.4.1. Description of the Human Mutations

The L285P (T4160C) mutation in ND1 is found in the eighth and last transmembrane helix, near the matrix side. It is not in contact with any other subunits and is buried within the bundle of ND1 transmembrane helices. The residue Y277C (A4136G) is found at the matrix side of the seventh transmembrane helix, near to L285. It is in contact with a helical segment of NDUFS8 (NuoI), near its N-terminus, but distant from its two FeS clusters. L285P was modeled in the *nuoH* gene as V297P. Y277C was modeled as L289A and L289S. The locations of these residues for the human and the *E. coli* proteins are shown in Figure 6. The ND1_L285P (T4160C) mutation was first discovered [56] in a Queensland family with LHON that was originally identified by Wallace [57]. It was reported that individuals carrying a second mutation, Y277C (A4136G), experienced milder symptoms, suggesting an interaction between the residues.

#### 3.4.2. Effect of Substitutions on Function of Complex I

**Figure 7:**
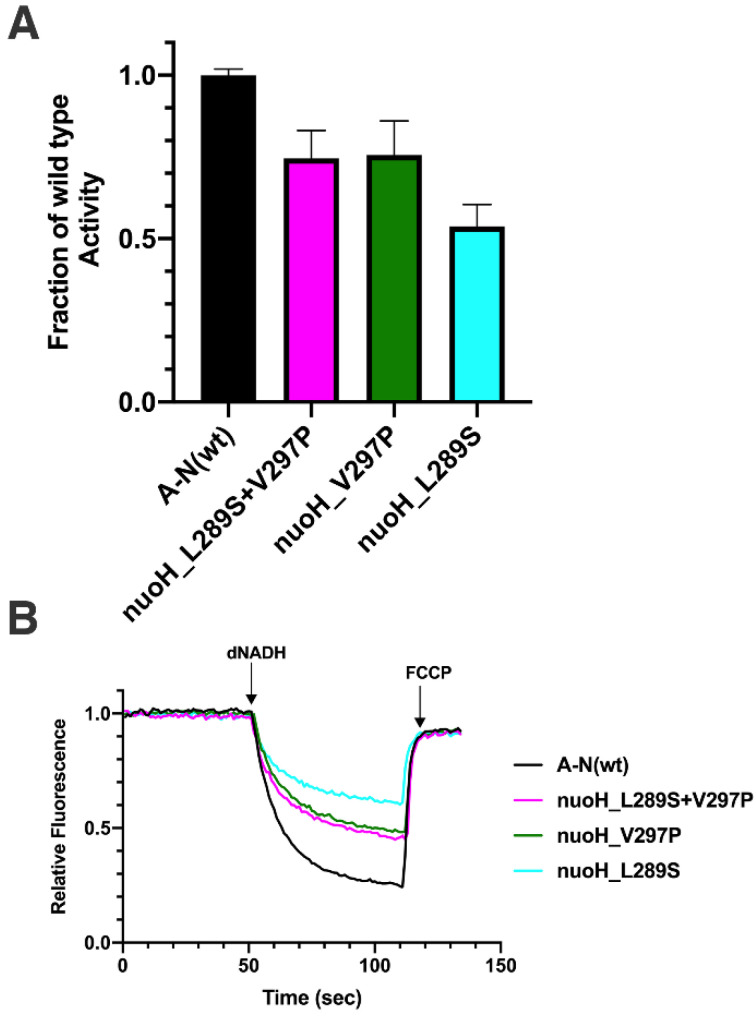
Effect of L289S on the activity of V297P in *E. coli* Complex I. L289S and L289S/V297P were expressed from vector pBAD33(A-N), and membrane vesicles were prepared. (**A**) dNADH oxidase activity of the membranes was normalized to the value of activity from the wild type pBAD33(A-N). Results are the means and standard deviations from 2–3 membrane preparations with 4 replicates each. (**B**) Proton translocation activity of Complex I.

In *E. coli*, mutants V297P, L289A, L289S, and L289S + V297P were constructed in *nuoH* and moved to the expression vector pBAD33(A-N). Membrane vesicles were prepared, and enzyme activity assays were measured. Complex I activities for NuoH_L289A and L289S are shown in Figure 5A. L289A had an activity of 69 ± 5% of wild type, while L289S had a somewhat lower level of 53 ± 6%. The rates of dNADH oxidase for V297P, L289S, and L289S + V297P are compared in Figure 7A. The activity of NuoH_V297P was 75 ± 8%, nearly identical to that of the double substitution, L289S + V297P, at 75 ± 8%. Since the single substitution L289S had an activity of 53 ± 6%, the activities are not additive, and this suggests an interaction between the residues. A similar interpretation can be reached by examining the rates of proton translocation (Figure 7B). Both L289 variants showed a band for NuoH in the immunoblot (Figure 5C). In the native gels (Figure 5D,E), both were similar to the wild type.

## 4. Discussion

In this report, a broad range of clinical mutations of ND1 was analyzed in a bacterial model system. The use of an HA-tagged NuoH allowed the expression of this subunit to be determined. In conjunction with native gel analysis for determination of assembly, and activity measurements for functional analysis, a detailed picture was obtained for each mutant. The results of each can be described as fitting into one of four categories: (1) Lack of expression of NuoH, hence no assembly or activity. (2) Normal or near-normal expression of NuoH, but low levels of assembly and activity. (3) Normal expression of NuoH and assembly of Complex I, but very low levels of activity. (4) Normal levels of expression and assembly, and moderate levels of activity, e.g., ~50–75% of wild type. See Table 1 for a summary of the human mutants.

**Figure 8:**
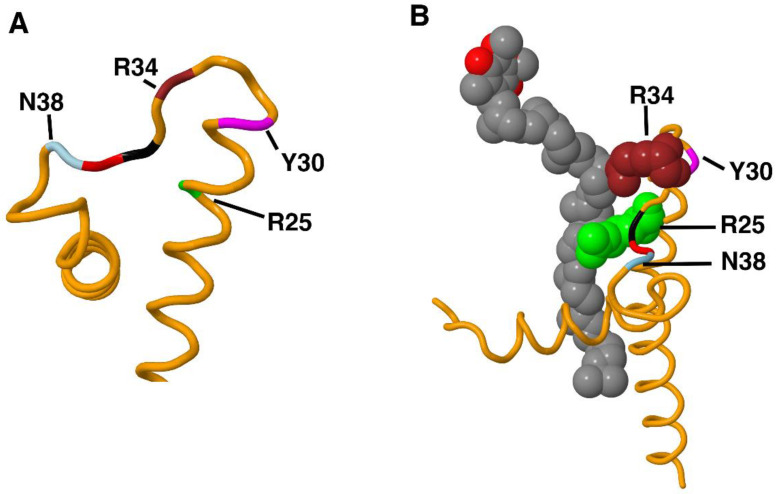
Structural context of R25, Y30, R34 and N38 in ND1. The structure of porcine ND1 was used for its inclusion of bound ubiquinone. (PDB file: 7V2C). (**A**) The trace of ND1 shows the highly twisted loop connecting the two helices at the N-terminus of ND1. Residue R25 is colored lime, Y30 is magenta, R34 is brown, and N38 is light blue. Residues 36 and 37 are colored black and red, respectively. (**B**) Bound ubiquinone, shown in space-filling view, is contacted by both R25 (lime) and R34 (brown). The side chains of both N38 and Y30 are pointed in the opposite direction, away from the ubiquinone.

Both NuoH_N50A and N50D are in category (1), in which there is little or no expression of the subunit, and so no assembly or activity. This mutation, ND1_N38D (A3418G), was “Reported” in MitoMap, while in ClinVar the mutation is not listed. In the original study [54], no information is reported about the expression or activity of Complex I. Insight might be obtained by considering the structure of the subunit in the region of the mutation. The human sequences (residues 22–57) are highly conserved with the *E. coli* sequence (36–71): 42% identical. As shown in Figure 8A, the conserved sequence containing N38, GPN, colored black, red, and light blue, begins a highly twisted segment that connects a longer transmembrane helix and a shorter helix. It seems plausible that an amino acid substitution in this region could disrupt folding of ND1 (NuoH), preventing assembly and leading to degradation of the subunit. A similar outcome was seen with NuoH_R46H, which had poor expression of subunit H and essentially no assembly of Complex I. It is shown colored brown in Figure 8A as ND1_R34H and is located in the same region as ND1_N38D. At MitoMap it is described as “Conflicting Reports”, and it is not listed in ClinVar. When this mutation was discovered in a 6–10 year-old boy with indications of mitochondrial disease, enzyme deficiencies were seen with Complexes II and III, but not Complex I [53]. In Figure 8B, it can be seen that R34 contacts the ubiquinone [24], providing a potential reason why some mutations are particularly deleterious. The alanine substitution in NuoH_R46A was somewhat different in that the expression and assembly were both normal, while the activity was very low (about 2% of wild type). Using a different *E. coli* expression system [61], it was shown that NuoH_R46A had an activity that was 13% of wild type. They also found that R46K had an activity of 46%, further supporting that the outcome is dependent upon the substituted amino acid. In a human Complex I structure lacking a bound quinone, PDB file 5XTD, [62], this arginine is part of a hydrogen-bonding network involving NDUFS7_D102 and D104, and ND1_R25 [62]. In the bacterial enzymes, the situation is slightly different. D104 is not conserved in either bacterial enzyme, but *T. thermus* has a second aspartic acid, D55, upstream from the conserved aspartic acid, D59. There is not a second aspartic acid in this region of NuoB in *E. coli.* The results of analysis using the *E. coli* enzyme show that some substitutions of NuoH_R46 are more deleterious than others. In the case of ND1_N38, the conformation of the loop where this residue resides might make many different substitutions deleterious.

In the second category are NuoH_E71K and E228K, corresponding to human ND1_E59K and E214K, respectively. Both seem to have normal expression of NuoH, but assembly of Complex I in the native gel appears less than wild type. The activities of NuoH_E71K and E228K are very low: 7% and ~0, respectively, suggesting that the assembled Complex I is largely inactive. Both mutants are not found in ClinVar, and at MitoMap they are listed as “Reported”. In humans, ND1_E59 is buried between subunits NDUFS7 (NuoB) and ND3 (NuoA), and is proximal to a conserved lysine residue, ND3_K33 in humans (NuoA_K46 in *E. coli*). Therefore, it can be seen why the alanine substitution is more tolerated for assembly than is lysine, while the activity is still low, 15%. Using a different expression system in *E. coli*, activity of the alanine mutant was reported to be somewhat higher, 48% [61]. In the clinical analysis [58], ND1_E59K showed evidence of assembly, but had very low Complex I activity, which seems consistent with the bacterial results in this report. In the case of ND1_E214K, it was reported that the activity was low in both muscle and fibroblasts, and that the level of assembly seen in native gels was also low [59]. This mutation was discovered more recently in a screen of mitochondrial disease patients [63]. The very low levels of Complex I activity seen in the bacterial system, NuoH_E228a and E228K, can be rationalized on the basis of interactions with NuoB (FS7). In humans, it appears that residue E214 is ion-paired with a conserved arginine in NDUFS7, R115. In *E. coli*, it is NuoB_R91. Therefore, we suggest that the lysine substitution affects the assembly of Complex I; any mutation could disrupt function because the terminal FeS cluster is in NDUFS7 (NuoB); and ND1 (NuoH) is important for ubiquinone binding. This is supported by various results of mutagenesis in *E. coli.* From a previously cited study [61], an activity of 9% was found for NuoH_E228A, compared to 1% here. They also analyzed the E228Q mutant, as did another group [64], and both found about 1% activity for the isosteric but non-ionizable glutamine substitution. Similar to our results, the latter group reported 2% activity for NuoH_E228K and additionally, 47% for E228D. It was previously suggested that the ND1_E214K mutation would disrupt coupling between quinone reduction and proton translocation [65].

The third category includes NuoH_R37A and Q (ND1_R25A and Q), as well as the previously discussed alanine substitutions NuoH_R46A, E71A, and E228A. For these five variants, expression and assembly appear to be normal, but activity is near zero. The ND1_R25Q mutation was found in a 62-year-old woman with late-onset MELAS [45]. The mutation was heteroplasmic, 50% in muscle, with elevated levels of ragged-red fibers. All rates of electron transport were in the normal range, including Complex I. This mutation was described as “Pathogenic” at ClinVar, but only “Reported” at MitoMap. ND1_R25 is found in the same hydrogen bonding network as ND1_R34, with two aspartic acid residues in NDUFS7: D102 and D104. In *E. coli* we found that the effect of these mutations on activity were significant. NuoH_R37A was evaluated previously and found to have 9% of the activity [61]. In humans, Complex I activity was in the normal range, but these measurements are difficult to evaluate due to heteroplasmy and the uncertainty of a proper control activity. The location of ND1_R25 relative to the N2 FeS cluster and the ubiquinone-binding site suggests that mutations could disrupt binding and reduction of the quinone. Contacts between ND1_R25 and ubiquinone are shown in Figure 8B, along with ND1_R34.

In the fourth category are the mutants in which expression and assembly appear normal, and the activity is substantial, approximately in the range of 50–75% of wild type. The first example is NuoH_R291Q, which had 50% of wild type activity. The corresponding human mutation, ND1_R279Q, identified by high-throughput DNA sequencing [55], was reported in ClinVar as “Likely pathogenic” and in MitoMap as “Reported”. It was revised from a “Variant of Uncertain Significance” to “Likely Pathogenic” in a recent evaluation [18]. A related mutation, ND1_R279L, was discovered in a four-year-old with Leigh Syndrome, with reduced Complex I activity [66]. In *E. coli*, the alanine substitution had a similar activity to the glutamine, 55%, suggesting that the loss of the original arginine was key. In the human enzyme, R279 is near subunit NDUFS2, but its primary contacts are in ND1. The nearby arginine R281 hydrogen bonds with NDUFS2_D446. There are three conserved glutamic acid residues in this region of ND1: E202, E204, and E206A. The third one, E206, hydrogen bonds to R279. A similar situation is seen in the *T. thermus* enzyme, but in *E. coli*, the conformation of NuoH is quite different in this region of the recent structure. The side chain of residue R291, corresponding to ND1_R279, is not resolved, but R293 is hydrogen-bonded to E218 of NuoH, which corresponds to the middle glutamic acid, ND1_E204, and not to a residue in NuoCD. It is not clear if this reflects a mechanistic reshuffling of interactions. What is clear is that it would be difficult to predict the consequences of mutations in this region. These relatively high activities seen after substitution of the arginine (NuoH_R291) suggest some redundancy in the interactions important for conformational changes.

The results of NuoH_L42A and S also fall into category 4, in which expression and assembly appear normal, and activity is in the range of 50–75%. A drawback of this analysis is the lack of conservation at this site, tyrosine in humans and leucine in *E. coli*. To avoid possible complications from the introduction of cysteine, in this study serine was selected instead. The ND1_Y30C mutant was discovered first in individuals that carried other mutations in mitochondrial DNA [46,47]. Later it was identified without other mutations, but was not further characterized [67], and again, when it was characterized only phenotypically [50]. The latter report concluded that the cysteine probably played a role in the phenotype and that the genetic background in which this mutation occurs may be influential. Prior to this report, it was classified as “Likely benign” at ClinVar, and “Reported” at MitoMap, and in 2020 was downgraded to “Benign” after a re-evaluation [18]. Both ND1_Y30C (G3395A) and ND1_Y30H (T3394C) have been discovered in various populations [49,68] and with various disease states [69,70]. In the human enzyme, ND1_Y30 hydrogen bonds with NDUFA1_E4, a supernumerary subunit. Two lysine residues from ND1 are nearby, K26 and K35. Mutations in this region were predicted to affect quinone entry to the site of reduction [65], and that might be reflected in the reduced rates of enzyme activity.

The mutants ND1_Y277C (A4136G) and ND1_L285P (T4160C) can be placed in category four, but they have also been suggested to have interacting or suppressor-like effects when appearing together [56]. In a well-documented family with a high prevalence of LHON, these two mutations were first identified together [57]. In one branch of the family, all nine members were homoplasmic for ND1_L285P, while five also contained the second mutation, ND1_Y277C [56]. Those with both mutations appeared to have milder symptoms and higher levels of Complex I activity [71]. To complicate the interpretation, all members appeared to carry the confirmed LHON mutation ND6_M64V (T14484C). In two other reports, individuals with ND1_Y277C (A4136G) also carried a different confirmed LHON mutation, ND4_R340H [72,73]. More recently, new insights were obtained [60]. A 19-year-old male was identified with both mutations, ND1_Y277C (A4136G) and ND1_L285P (T4160C), but his mother and sister carried only the ND1_Y277C (A4136G) mutation. All were homoplasmic for the ND1_Y277C (A4136G) mutation. The male was heteroplasmic for the other mutation and at high levels of 80–90% in blood, urine sediment, and buccal mucosa. He was diagnosed with LHON plus dystonia, while the mother and sister were asymptomatic. Biochemical analysis showed that levels of Complex I proteins were reduced in the male, along with Complex I activity, suggesting that the ND1_L285P (T4160C) mutation was pathogenic in this background. Since this mutation could not be analyzed in the absence of the ND1_Y277C (A4136G) mutation, a full interpretation is not yet possible. When this mutation was analyzed in *E. coli,* NuoH_V297P, both here at 76%, and in a prior study at 62% [74], the effect on activity was mild. These two studies diverged on the outcome of the double mutant. Here, the second substitution NuoH_L289S had a lower activity, 53%, and the double substitution was 75%, essentially the same as the single mutant NuoH _V297P, 76%. In other words, the first mutation was a suppressor of the second mutation. In the prior study, where the NuoH _L289C substitution was made, the effects of the two mutants were additive, with the double mutant having lower activity than either single mutant. Our conclusion is that suppressor effects may be difficult to capture in a bacterial model system, especially where the sequence conservation is not sufficiently high.

In summary, the rapidly increasing number of structural studies by cryo-EM have provided new insights into mitochondrial Complex I. New bacterial Complex I structures and the ability to track NuoH (ND1) by immunoblotting have improved the modeling of mitochondrial mutations in *E. coli* and their interpretations. All of the mutations examined in this study showed significant losses in activity. In four of the nine mutants, assembly was disrupted, consistent with residues located at subunit interfaces. We conclude that a bacterial model system can provide useful insights about mutations in mitochondrial genes, not only existing mutations, but those discovered in the future. One requirement for the ultimate goal, the evaluation of all mitochondrial gene variants, would be the analysis of significant numbers of benign mutations in the bacterial system. In this way the model would be more rigorously tested with both positive and negative controls. The lack of benign variants in this study is a noted limitation. The same approach can be used to analyze mutations that occur in mitochondrial genes responsible for Complex III, Complex IV, and Complex V (ATP synthase). We conclude that a bacterial model system can provide useful insights about mutations in mitochondrial genes, not only existing mutations, but those discovered in the future. One requirement for the ultimate goal, the evaluation of all mitochondrial gene variants, would be the analysis of significant numbers of benign mutations in the bacterial system. In this way the model would be more rigorously tested with both positive and negative controls. The lack of benign variants in this study is a noted limitation. The same approach can be used to analyze mutations that occur in mitochondrial genes responsible for Complex III, Complex IV, and Complex V (ATP synthase). Indeed, work has already been reported for the ATP synthase along these lines [75,76]. Since *E. coli* lacks a homologous Complex III or IV, mitochondrial mutations in those enzymes must be analyzed in other bacteria. Indeed, work has already been reported for the ATP synthase along these lines [75,76]. Since *E. coli* lacks a homologous Complex III or IV, mitochondrial mutations in those enzymes must be analyzed in other bacteria.

## Figures and Tables

**Table 1:** Summary of Human ND1 Mutants.

Category *^1^*	Human Mutation	Analysis of Human Complex I	*E. coli* Substitution	Expression of *nuoH ^2^*	Assembly of Complex I *^3^*	dNADH Oxidase Activity *^4^*
1	N38D (A3418G)	Disrupted assembly [54]	NuoH_N50D	-	-	<1%
1	R34H (G3407A)	Complex I activity was in the normal range [53]	NuoH_R46H	-	-	4%
2	E59K (G3481A)	Very low Complex I activity [58]	NuoH_E71K	+	+/−	7 ± 3%
2	E214K (G3946A)	Very low Complex I activity [59]	NuoH_E228K	+	+/−	<1%
3	R25Q (G3380A)	Complex I activity was in the normal range [45]	NuoH_R37Q	+	+	<1%
4	R279Q (G4142A)	No information on Complex I activity [55]	NuoH_R291Q	+	+	50 ± 7%
4	Y30C (A3395G)	Decrease in quantity and activity of Complex I, but no sub-assembly was detected [50]	NuoH_L42S	+	+	66%
4	Y277C (A4136G)	Asymptomatic in homoplasmic individuals [60]	NuoH_L289S	+	+	53 ± 6%
4	L285P (T4160C)	Was not analyzed in the homoplasmic state.	NuoH_V297P	+	+	76 ± 10%

*^1^* Based on expression, assembly, and activity: see Discussion for details. *^2^* Immunoblotting of membrane fraction. *^3^* Estimation from native gel electrophoresis, +/− signifies very low assembly. *^4^* Activity in membrane vesicles compared to wild type Complex I.

## Data Availability

Not applicable.

## Supplementary Materials

The following supporting information can be downloaded at: https://www.mdpi.com/article/10.3390/life12111934/s1.
