# Supplementary material for: Analysis of Human Clinical Mutations of Mitochondrial ND1 in a Bacterial Model System for Complex I
Source: Life (Basel). 2022 Nov 20;12(11):1934. doi: 10.3390/life12111934 (PMC9696053; doi:10.3390/life12111934)
Supplement: Supplementary file 1 [file life-12-01934-s001.zip › Supplementary Table S1.pdf]

Supplementary Table S1
Primers used for Q5 mutagenesis

Mutation[1]	Restriction Sites[2]	Forward primer	Reverse primer
R37A	GGCGCGCC<br>Asc I	GCTTTGGCGAGGCGCGCCTGCTGGG	TCATGAATGCCCCGCAGG
R37Q	AGCGCT<br>Afe I	TTTGGCGAACAGCGCTTGCTGGGTCTG	GCTCATGAATGCCCCGCA
L42A	GGCGCC<br>Kas I	GCCTGCTGGGCGCCTTCCAGAACCGTTAC	GACGTTCGCCAAAGCTCA
L42S	CYCGRG<br>Ava I	TCGTTCCAGAACCGTTACGGACCTAAC	CCCGAGCAGGCGACGTTCGCCA
R46A	CGTACG<br>BsiW I	GTTCCAGAACGCGTACGGACCTAACCGT<br>GTTGGCTG	AGACCCAGCAGGCGACGT
R46H	CCA-N_9-<br>TGG<br>Xcm I	TTCCAGAACCATTATGGACCTAACCGTGT<br>TG	CAGACCCAGCAGGCGACG
N50A	GCCGGC<br>NgoM I	ACCGTTACGGGCCGGCCCGTGTTGGC	TCTGGAACAGACCCAGCA
N50D	CGATCG<br>Pvu I	CGTTACGGACCCGATCGTGTTGGCT	GTTCTGGAACAGACCCAG
E71A	Eci I<br>GGCGGA	TGTTCTTTAAGGCGGACTGGATCCCG	TTTTGATCATGTCCGCAAC
E71K	Loss of Dra I<br>TTTAAA	TGTTCTTTAAGAAGGACTGGATCCCG	TTTTGATCATGTCCGCAAC
E228A	CTRYAG<br>Sfc I	TAGCGGTATGAAGTTCGGTCTG	TAGGCAATGTGGTAACCATCCG
E228K	AGTACT<br>Sca I	TTACCACATTAAGTACTCCGGTATGAAGT<br>TCGG	CCATCCGCCAGTTCCTGC
L289A	CCGCGG<br>Sac II	GTATGACCAGGTAATGTCCTTCGGCTGGA<br>AA	CGCGGACGCGGCGCCGACGCACGAATCA<br>AAATG
L289S	CCGCGG<br>Sac II	GTATGACCAGGTAATGTCCTTCGGCTGGA<br>AA	CGCGGACGCGGCGACGACGCACGAATCA<br>AAATG
R291A	CCGCGG<br>Sac II	GTATGACCAGGTAATGTCCTTCGGCTGGA<br>AA	CGCGGCGCCGGTAACGACGCACGAATCA<br>AAATG
R291Q	CCGCGG<br>Sac II	GTATGACCAGGTAATGTCCTTCGGCTGGA<br>AA	CGCGGTTGCGGTAACGACGCACGAATCA<br>AAATG
V297P	CCGCGG<br>Sac II	GTATGACCAGCCAATGTCCTTCGGCTGGA<br>AA	CGCGGACGCGGTAACGACGCACGAATCA<br>AAATG

[1] Codons for the mutated residues are colored red
[2] Restriction sites used for identification of mutations are colored blue (unless the nucleotides include the mutated residue)
